# Features extraction from multi-spectral remote sensing images based on multi-threshold binarization

**DOI:** 10.1038/s41598-023-46785-7

**Published:** 2023-11-11

**Authors:** Bohdan Rusyn, Oleksiy Lutsyk, Rostyslav Kosarevych, Taras Maksymyuk, Juraj Gazda

**Affiliations:** 1 Department of Remote Sensing Information Technologies, Karpenko Physico-Mechanical Institute, NAS of Ukraine, Lviv, Ukraine; 2 Department of Informatics and Teleinformatics, Kazimierz Pulaski University of Technology and Humanities, Radom, Poland; 3 Department of Telecommunications, Lviv Polytechnic National University, Lviv, Ukraine; 4 Department of Computers and Informatics, Technical University of Kosice, Kosice, Slovakia

**Keywords:** Computer science, Computational science, Ecology

## Abstract

In this paper, we propose a solution to resolve the limitation of deep CNN models in real-time applications. The proposed approach uses multi-threshold binarization over the whole multi-spectral remote sensing image to extract the vector of discriminative features for classification. We compare the classification accuracy and the training time of the proposed approach with ResNet and Ensemble CNN models. The proposed approach shows a significant advantage in accuracy for small datasets, while keeping very close recall score to both deep CNN models for larger datasets. On the other hand, regardless of the dataset size, the proposed multi-threshold binarization provides approximately 5 times lower training and inference time than both ResNet and Ensemble CNN models.

## Introduction

Remote sensing tasks are currently vital for a wide range of applications in our life. The workflow of remote sensing is typically connected to the analysis of large data flows by expert systems and deriving the corresponding inferences, evaluations and predictions. Naturally, such workflows heavily rely on artificial intelligence (AI) solutions^1^. By using deep learning (DL), modern AI algorithms achieved a significant boost in terms of speed and accuracy of decision making, as well as in terms of the overall volume of the processed data in real time^2, 3^. Notwithstanding the already terrific abilities of state-of-the-art AI-empowered remote sensing solutions, which are able to process images in quasi-real-time with 15–20 fps, there is still a large subset of tasks that require processing time in the range of milliseconds^4^. The most important aspect of the computational performance of the deep learning algorithm is the feature extraction process, which significantly affects the classification performance^5^. According to the literature, feature extraction typically takes up to 80% of all computational loads^6^. The most widely adopted DL solution for image and video processing is based on convolutional neural networks (CNNs), which use a mix of convolutional and dense (fully connected) layers^7, 8^. The convolutional layers work as feature extractors, while dense layers are used for classification or regression decisions based on the extracted features. Sometimes, feature extractors can use recurrent layers, which provide generative capabilities to the model. Thus, the internal structure and deepness of the CNN model is usually optimized for the specific task to achieve the best performance in terms of accuracy, precision, sensitivity and inference time^7, 8^.

By nature, deeper CNNs are typically more powerful and can be used for more complex tasks in remote sensing. However, the size of the CNN, or any other DL model, is directly connected to the computational requirements and processing time, which constrain its practical applicability. Thus, the alternative approach is to use models based on binary neural networks^9^, i.e., neural networks, where all weights are binary, instead of floating or integers. Such an approach allows the use of much simpler hardware and provides much faster inference on segmentation tasks based on the generalization of image features^10^.

Typically, CNNs are very likely to run into overfitting by lacking generalization abilities and being biased to the training data. There are several techniques to avoid overfitting, such as adding additive noise to the training data, using dropout layers and optimizing the ratio between model complexity, number of training samples and target accuracy^11–14^. Feature extraction is a far more complicated process than classification and requires additional optimization steps for training^15^. The training time for the model depends on the internal number of trainable parameters and the size of the dataset. Therefore, the initial optimization direction is to determine the optimal dataset, which is relatively small but provides the target variability, discriminative ability and statistical completeness^16^.

The distinctive property of the typical remote sensing task compared to conventional computer vision tasks is the multi-spectral image processing^17^. Multi-spectral images are composed of a set of monochrome subimages of the same scene from different sensors (cameras) with different wavelengths. The commonly used RGB images are the particular case of the multi-spectral image composed of subimages of red, green and blue colors. Industrial remote sensing satellites, such as Landsat 5, work with 7 subimages, which cover both the visible and infrared ranges from 450 to 1250 nm.

All known approaches for image processing and recognition can be applied for the multi-spectral images by processing each subimage separately^18^. For example, the multi-spectral image can be considered a combination of monochrome images, which are processed separately to extract the distinctive features and landmarks and combine them within a single image. However, better results can be achieved by multi-spectral processing of the image as a whole. This is especially important for the classification tasks, when we need to extract the feature vectors and create a dataset for deep learning^19^. To fully exploit the additional information from several spectral bands, we need to analyze a multi-spectral image as a whole rather than a combination of multiple grayscale images. Thus, each pixel of a multi-spectral image can be represented in a n-dimensional hyperspace as a vector of length k.

There are specific methods dedicated to the processing of the multi-spectral images. In the segmentation of multi-spectral images^20^, each pixel in a different spectrum band forms a vector of features with different intensities, which represent its position in the k-dimensional feature space. The simplest approach to determine a class is to choose the upper and lower thresholds for each spectrum band to represent an m-dimensional hypercube in a feature space. If the feature vector of the pixel fits into the corresponding class localization in a hypercube, the pixel is classified to that class accordingly. In many tasks, segmentation can be simplified to binarization, which reduces the computational complexity and uncertainty of decisions^21, 22^.

Usually, the classification of the multi-spectral images by deep learning methods is implemented in the same way for any problem. The key difference is all the time hidden in the feature extraction part, which is implemented by the convolutional neural networks^23, 24^. As an alternative, multi-level approaches are sometimes used, such as decision trees and approaches based on gradient boosting^25^.

From the perspective of processing and feature extraction of multispectral images, decomposition methods have proven to be effective, as they enable the detailing of potential hidden features^26^. In particular, approaches utilizing processing in the spectral domain^27^ and methods based on the use of subpixel data^28^ for multispectral images can be distinguished. Feature formation technology based on threshold approaches works well for multichannel images^29^, making it possible to apply it to multispectral images.

In the majority of cases, approaches to feature extraction of multispectral images rely on local descriptor methods that do not account for possible relationships in homogeneous regions. To address this issue, a method is proposed in^30^ that is based on the extraction of structural features with multiple characteristics of spectral-spatial structures of different objects. Here, local and global structural features are formed and combined into a general feature vector.

In^31^, it is further suggested to utilize multiscale features, which enable effective representation in the feature space of image regions containing objects of arbitrary size or varying scales, thereby enhancing the ability to analyze multispectral images at different levels of detail. This approach allows for the efficient handling of multispectral images, catering to the diverse requirements of various applications in the realm of remote sensing, pattern recognition, and image processing.

The main problem, that occurs during the recognition of multi-spectral images, is that the overall data volume for processing is significantly higher, which negatively affects the processing time and memory consumption. In many tasks, it results in significant constraints for real-time computer vision applications. Recently, we observe numerous developments of smaller and faster CNN architectures such as MobileNet, SqueezeNet, ShuffleNet, etc, which are suitable for resource-constrained environments, such as microcontrollers and mobile devices. However, the main limitation of those models is that they still use convolutional layers for the feature extraction, but their number is significantly reduced and some layers may be simplified by replacing regular convolutions with depthwise convolutions. With depthwise convolution we separate all channels of the image, perform independent convolutions for each channel, and stack the result afterwards. This approach has been proven to be effective for many tasks on RGB images, where number of channels is limited to 3. Typically, those models are just 1–5% below their much larger counterparts in the key performance metrics^32^. However, multi-spectral images sometimes can exceed 100 contiguous spectral bands (channels), which makes depthwise convolutions less favourable in this context. Moreover, a variable number of channels in multi-spectral images does not align well with highly determined architecture of any CNN, where even small change of image resolution has large impact on the overall network performance. Thus, such architectures are not yet suitable for the remote sensing applications.

Therefore, further research on feature extraction approaches for multi-spectral images to address real-time requirements is timely and relevant to the overall field of deep learning-based computer vision and remote sensing applications in particular.

To address the aforementioned problem, in this paper we propose a new approach for the features extraction from multi-spectral images. Proposed approach is aimed to decrease the complexity of the computer vision applications in remote sensing. In particular, we assess the training complexity of the deep learning models on multi-spectral images due to the convolutional layers. We also prove that employing preliminary feature extraction, based on multi-threshold binarization, allows to speed-up the model training process. In addition, the proposed approach is considerably faster and requires a smaller training set compared to conventional training of the convolutional neural networks. The main contribution of the paper are the following:

1. We develop a new feature extraction algorithm from multi-spectral remote sensing images based on the multi-threshold binarization.
2. We derive a mathematical model for calculation of an arbitrary number of thresholds for image binarization.
3. We conduct an experimental evaluation and comparison between proposed approach and commonly used CNN models.

The remainder of this paper is organized as follows. In section “Introduction”, we explain in detail the proposed approach for feature extraction from multi-spectral images based on the multi-threshold binarization. In section “Multi-threshold binarization for feature extraction from multi-spectral images”, we provide an experimental evaluation and performance analysis. Finally, we conclude this article in section “Experimental evaluations and performance analysis”.

## Multi-threshold binarization for feature extraction from multi-spectral images

Remote sensing images often contain noise and artifacts. To remove or mitigate this noise, we propose to use a multi-threshold image binarization, as it allows for the filtering of pixels that do not meet specified threshold values. Multi-threshold binarization is a powerful technique, that performs an adaptation of thresholds for each image or for distinct regions within an image, thereby facilitating enhanced feature extraction under various conditions. If it is necessary to highlight objects or features in an image that possess varying contrast or brightness relative to the background, multi-threshold binarization can aid in enhancing extraction accuracy by utilizing different thresholds for different parts of the image.

Thus, the design of the feature extraction algorithm should satisfy the following requirements:

- distinguish foreground objects under different lighting and brightness conditions;
- support smooth and sharp contours;
- be robust to the additive noise;
- be invariant to affine transformation;
- be invariant to nonlinear distortions;
- be compliant with real-time operation.

The conventional way of binarization of grayscale and color images is to use a global threshold to simply obtain a map of the binary features:

1 $$\begin{aligned} \left\{ \begin{array}{ll} B(x,y)=0,&{}\quad if\,\, f(x,y)<T \\ B(x,y)=1,&{}\quad if\,\, f(x,y) \geqslant T \end{array} \right. \end{aligned}$$

where *B*(*x*, *y*) is a binary representation, *f*(*x*, *y*) is a grayscale image, *T* is binarization threshold.

However, considering that deep dense neural network (DNN) is used as a classificator in modern computer vision applications, the number of features, that are extracted with the global threshold binarization is not sufficient for the accurate classification of multi-spectral remote sensing images. Therefore, in this paper we propose a multi-threshold binarization, which allows to transform each subimage into multiple binary representations based on different thresholds. The corresponding set of binary representations is treated as an informative tensor of features for each subimage. The key advantages of the proposed approach are that it allows to extract more features, and also provides a flexibility of feature extraction by using variable number of thresholds.

Let us describe the workflow of the proposed approach. Initially, the multi-spectral image is analyzed pixelwise, considering all spectrum bands, which are represented as a set of matrices. Then, we determine the global threshold, which can be calculated as an average of all values:

2 $$\begin{aligned} {{T}_{G}}=1/{{n}_{G}}\underset{i=0}{\overset{{{n}_{G}}}{\mathop \sum }}\,{{P}_{i}}, \end{aligned}$$

where ${{P}_{i}}$ is the value of the pixel intensity, ${{n}_{G}}=x\times y\times m$ is a total number of all pixels, considering all spectrum bands m. Then, the upper and lower thresholds are determined from the maximal and minimal values of pixels intensity:

3 $$\begin{aligned} \begin{aligned} {{T}_{U}}=\max (I), \\ {{T}_{D}}=\min (I), \end{aligned} \end{aligned}$$

where ${{T}_{U}}$—upper threshold, ${{T}_{D}}$—lower threshold.

To determine the best trade-off between computational complexity and accuracy, we have conducted an experimental evaluation to assess the effective number of thresholds for each image. According to our observations on the studied datasets, the equilibrium is achieved at 7 local equidistant thresholds in combination with one global threshold:

4 $$\begin{aligned} \begin{aligned} {{T}_{1}}=\frac{{{T}_{G}}-{{T}_{D}}}{3}+{{T}_{D}}, \\ {{T}_{2}}=2\frac{{{T}_{G}}-{{T}_{D}}}{3}+{{T}_{D}},\\ {{T}_{3}}=8\frac{{{T}_{G}}-{{T}_{D}}}{9}+{{T}_{D}},\\ {{T}_{4}}={{T}_{G}},\\ {{T}_{5}}={{T}_{U}}-\frac{{{T}_{U}}-{{T}_{G}}}{3},\\ {{T}_{6}}={{T}_{U}}-2\frac{{{T}_{U}}-{{T}_{G}}}{3},\\ {{T}_{7}}={{T}_{U}}-8\frac{{{T}_{U}}-{{T}_{G}}}{9}.\\ \end{aligned} \end{aligned}$$

The further increasing of the number of threshold does not provide significant advantage comparing to the added computational complexity.

Let us generalize it to an arbitrary number of thresholds:

5 $$\begin{aligned} {{T}_{V}}=j\frac{{{T}_{U}}-{{T}_{D}}}{r-1}+{{T}_{D}}, \end{aligned}$$

where *V* is a threshold index, *r* is a number of thresholds.

**Figure 1:**
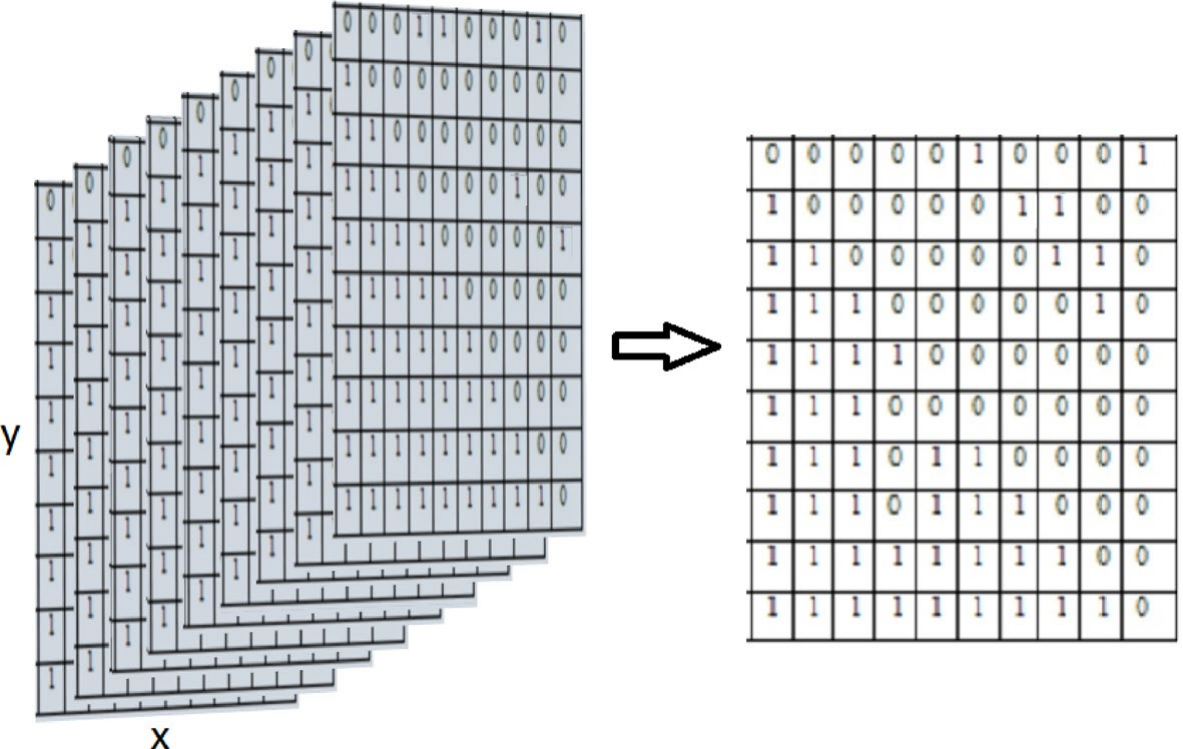
Representation of a separate subimage as a set of 9 matrices after multi-threshold binarization.

Multi-threshold binarization allows to represent each spectrum band of the multi-spectral image *I* as a set of binary matrices *L* (Fig. 1). Note that size of matrices ${{L}_{m}}\left( x,y \right)$ is the same as the size of the input sub-image$I\left( x,y \right)$.

Depending on the number of subimages *m* we will obtain a different number of matrices $L_{m}$.

To combine a large number of matrices, we use elementwise XOR and OR operations as follows:

6 $$\begin{aligned} L=\left( {{L}_{1}}\oplus {{L}_{2}} \right) \vee \left( {{L}_{3}}\oplus {{L}_{4}} \right) \vee \ldots \left( {{L}_{m-1}}\oplus {{L}_{m}} \right) , \end{aligned}$$

where *L* is a resulting binary matrix, which indicates the changes in particular elements of all matrices.

For the cases in which the invariance to rotations is not mandatory, matrix *L* is directly used as an input to the classifier, which can be either a classic one or based on the dense neural network. Whenever invariance to rotations is needed, we can use data augmentation to obtain a larger dataset with arbitrary image rotations.

A state-of-the-art remote sensing system consists of many sensors, which can determine an altitude, GPS coordinates and spatial orientation, represented as a quaternion:

7 $$\begin{aligned} q=a+bi+ci+dk, \end{aligned}$$

where *a*, *b*, *c*, *d*—real numbers, *i*, *j*, *k*—imaginary numbers.

A quaternion can be represented into Eulers angles according to the following equations:

8 $$\begin{aligned} \begin{aligned} \varphi&=\arctan \left( \frac{2\left( ab+cd \right) }{1-2\left( {{b}^{2}}+{{c}^{2}} \right) } \right) ,\\ \theta&=\arcsin (2\left( ac-db \right) ),\\ \psi&=\arctan \left( \frac{2\left( ac+bc \right) }{1-2\left( {{c}^{2}}+{{d}^{2}} \right) } \right) , \end{aligned} \end{aligned}$$

where $\phi ,\theta ,\psi$—rotation angles from the *x*, *y*, *z* axes, respectively.

The obtained angles $\phi ,\theta ,\psi$ can be used for the rotation of the feature matrix $L_{m}$ and perspective alignment. Thus, each sub-image $L_{m}(x,y)$ is rotated by the angle $\phi$. If the rotation is performed around the origin (0, 0), the corresponding transformation is represented according to the following equations:

9 $$\begin{aligned} \begin{aligned} {{x}_{2}}&=\cos (\varphi ){{x}_{1}}+\sin (\varphi ){{y}_{1}},\\ {{y}_{2}}&=-\sin (\varphi ){{x}_{1}}+\cos (\varphi ){{y}_{1}}, \end{aligned} \end{aligned}$$

where $\left( {{x}_{2}},{{y}_{2}} \right)$—coordinates after rotation. Considering that images can be obtained at different angles to the normal, we should also take into account a perspective transformation. This can be achieved by using rotations along the *y* and *z* axes:

10 $$\begin{aligned} \begin{aligned} {\textbf{R}}_{{\varvec{\theta }}}=\begin{bmatrix} cos(\theta ) &{}\quad -sin(\theta ) &{}\quad 0 &{}\quad 0\\ sin(\theta ) &{}\quad cos(\theta ) &{}\quad 0 &{}\quad 0\\ 0 &{}\quad 0 &{}\quad 1 &{}\quad 0\\ 0 &{}\quad 0 &{}\quad 0 &{}\quad 1, \end{bmatrix} \\ {\textbf{R}}_{{\varvec{\psi }}}=\begin{bmatrix} 1 &{}\quad 0 &{}\quad 0 &{}\quad 0\\ 0 &{}\quad cos(\psi ) &{}\quad -sin(\psi ) &{}\quad 0\\ 0 &{}\quad sin(\psi ) &{}\quad cos(\psi ) &{}\quad 0\\ 0 &{}\quad 0 &{}\quad 0 &{}\quad 1 \end{bmatrix} \end{aligned} \end{aligned}$$

where ${\textbf{R}}_{\varvec{\theta }},{\textbf{R}}_{\varvec{\psi }}$—rotation matrices along *y* and *z* axes.

By preprocessing the feature matrix $L_{m}$, we achieve invariance to the rotation and projection alignment. Such an approach provides faster training because it eliminates the need to train the model on an excessive dataset with additional rotated samples.

## Experimental evaluations and performance analysis

**Figure 2:**
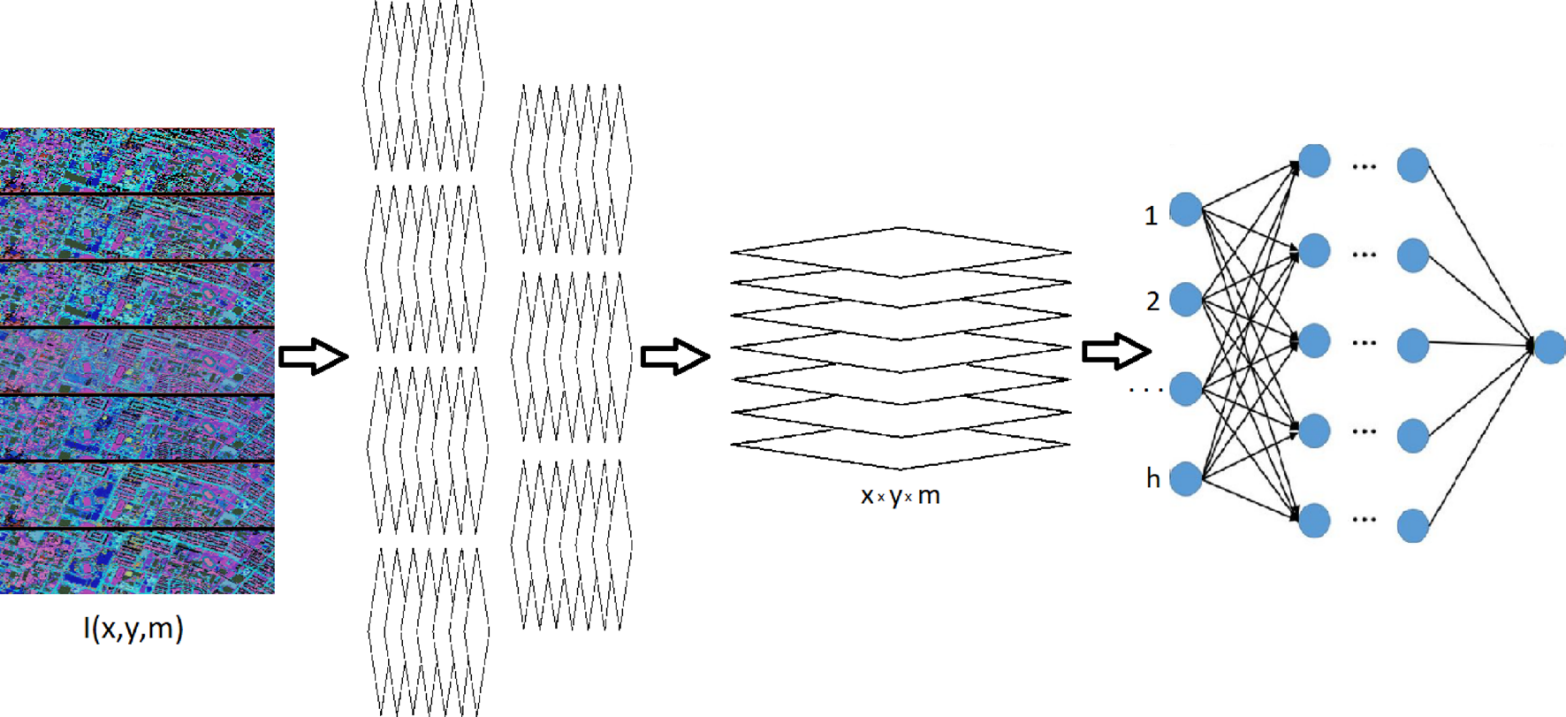
The workflow of the feature extraction from multi-spectral images.

The comparison of the proposed approach with existing ResNet and Ensemble CNN was conducted in terms of performance metrics and computational complexity during model training. Computational complexity significantly correlates with the training and inference time of the models. For this, ResNet, Ensemble CNN, and the proposed approach were trained from scratch on various sizes of training sets and with the same dense neural network head for all models. For each case, an assessment of validation accuracy and training time was conducted. The results showed that for some training set sizes, the proposed approach demonstrates the best classification accuracy results, while the performance of the proposed approach is almost 5 times higher. The experimental workflow is as follows. From each subimage of the multi-spectral image, we obtain the target number of binary representations for different binarization thresholds. The obtained representations are combined to create a vector of image features, which can be fed to the dense neural network classifier (Fig. 2). The invariance to rotations and affine transformations is ensured by the corresponding rotation operators and robust training. It is worth noting that dense neural network classifier, could have been replaced by other solutions, such as naive Bayesian classifier, support vector machines, random forest, k-nearest neighbors, etc. However, since our main aim is to compete with larger CNN architectures which all use dense neural network classifier, we also use it throughout this particular research to understand the exact impact of the proposed approach.

The optimal number of thresholds for each subimage of multi-spectral image is determined experimentally by looking at the classification accuracy. However, each additional threshold results in an increase in computational complexity and a longer inference time.

**Figure 3:**
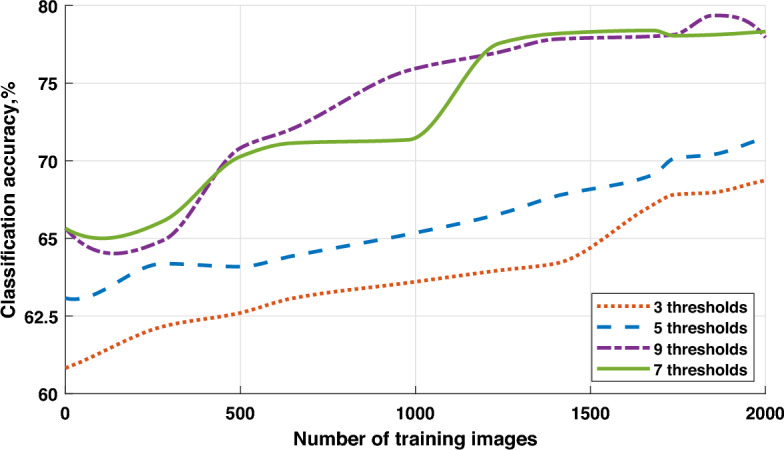
Classification accuracy vs number of thresholds for the Zurich dataset.

**Figure 4:**
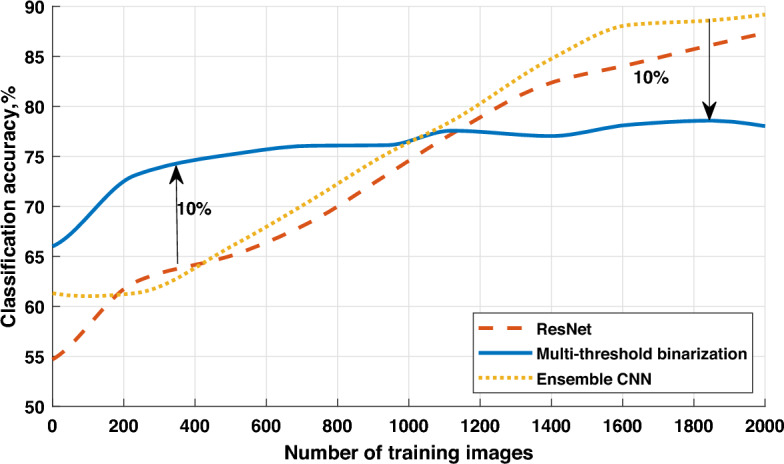
Classification accuracy vs dataset size for the TipJul1 database.

**Figure 5:**
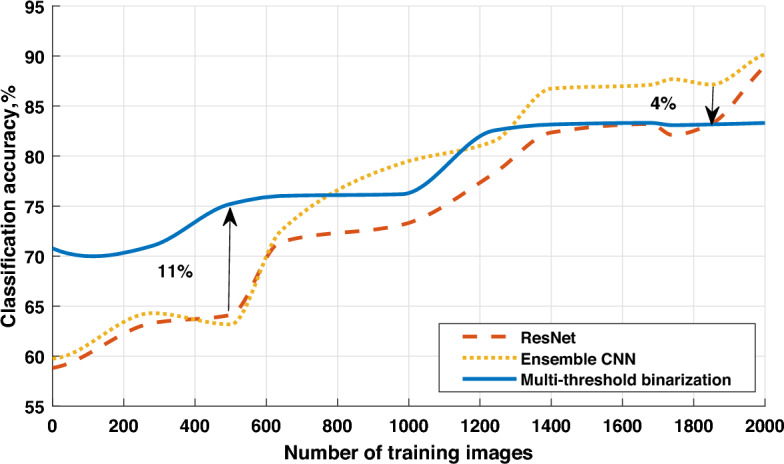
Classification accuracy vs dataset size for the Zurich database.

**Figure 6:**
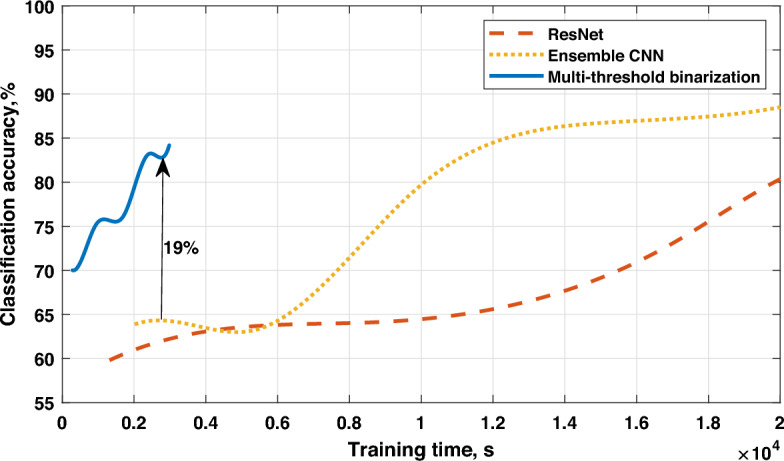
Accuracy vs training time for the Zurich database.

From the results in Fig. 3, we can conclude that the case with 7 thresholds satisfies most of the conditions, and further increasing of the threshold number does not necessarily improve the classification accuracy. To evaluate the performance of the proposed approach, we compare it with two reference architectures, namely, ResNet and Ensemble CNN. The dataset for the experiment is created based on the multi-spectral images obtained from remote sensing of the Earth’s surface. The whole dataset is split into 60%, 20% and 20% parts for training, validation and testing, respectively. The class labels are obtained from the homepage of the University of Valencia:

- The TipJul1 dataset based on Landsat data has 7 bands and 5 classes, with a resolution of 169x169 pixels;
- The Zurich dataset based on QuickBird data has 4 bands and 4 classes, with a resolution of 828$\times$889 pixels.

The ensemble of CNNs is based on the use of multiple CNN models, which are combined to create a single prediction. Ensemble learning is employed to enhance the performance of machine learning models by combining several models trained on the same training dataset. In this case, the ensemble is achieved by training multiple CNN models with the same architecture and parameters but with different initial weights. Predictions made by each of these models are combined based on the majority class with the highest number of votes. This combination of multiple models reduces the variance and increases the stability of predictions, leading to an improvement in classification accuracy.

From the results presented in Figs. 4 and 5, we can conclude that for small datasets, the proposed approach outperforms the ResNet and Ensemble CNN in terms of classification accuracy. This can be explained by the fact that both ResNet and Ensemble CNN extract features from the deep cascade of convolutional layers, which require large datasets for training. Thus, the key advantage of the proposed feature extraction approach is that it is able to work with a small training dataset, e.g. 200–800 images. Moreover, the proposed model still provides decent performance for large datasets (e.g. more than 1800 images), despite being outperformed with a small margin by deep CNN architectures in terms of classification accuracy. We further investigate the other performance metrics to compare the the proposed approach with two other models. In the Table 1 we depict the highest achieved values of accuracy, precision, recall and F1-score regardless of the training time and dataset size. All metrics have been measured for both TipJul1 and Zurich datasets. Obtained results correlate with those displayed on Figs. 4, 5 and 6, and we observe that multi-threshold binarization has noticeably lower scores in *accuracy*, *precision* and *F1-score*, while being very close to the more complex CNN models in *recall*. This can be explained by the difference between underlying meaning of those metrics. Whereas, *accuracy* and *F1-score* are the balanced metrics that reflect the models ability to classify true positive and true negative samples, the *precision* is biased towards minimizing false positive samples, and *recall* is biased towards minimizing false negative samples. In most computer vision tasks, we seek for a trade-off between *precision* and *recall*, reflected by the *F1-score*. However, in the context of many remote sensing applications, we often look for environmental anomalies such as large forest fires, erupting volcanoes, etc. Minimizing false negative samples allows to identify the problems earlier and save more time for a corresponding reaction. In this context, *recall* metric is the most important, which proves a suitability of the proposed approach specifically to the remote sensing computer vision applications. For a more comprehensive understanding, we also compare the training time for all studied models. To evaluate the training time of the models, standard tools contained in the TensorFlow deep learning library were used. In this case, for evaluating the ResNet and Ensemble CNN models, model training profiling tools were employed. The profiling log contains information about the execution time of the model operations; by analyzing it, one can determine how much time the training process takes. Assessing the training time of the proposed model was somewhat more complex since it involves the preliminary application of multi-threshold binarization. In this instance, checkpoint markers were used, and measuring the time between checkpoints allow to determine the training time. As observed from the results in the Table 2, the computational complexity of the multi-threshold binarization is much lower than those of ResNet and Ensemble CNN architectures, due to the absence of convolutional layers.

**Table 1:** Comparison of different performance metrics for the studied models.

Dataset	Model	Accuracy	Precision	Recall	F1-score
TipJul1	Multi-threshold binarization	0.77	0.46	0.94	0.62
	ResNet	0.88	0.64	0.97	0.77
	Ensemble CNN	0.86	0.60	0.95	0.74
Zurich	Multi-threshold binarization	0.83	0.53	0.96	0.68
	ResNet	0.87	0.59	0.97	0.73
	Ensemble CNN	0.86	0.57	0.96	0.72

**Table 2:** The relation between training time and the dataset size for the studied models.

Dataset size, images	Multi-threshold binarization, s	ResNet, s	Ensemble CNN, s
140	281	1316	2018
280	517	2874	4412
500	944	5307	8730
980	1708	9881	17,021
1400	2394	15,118	21,218
1740	2579	18,622	25,743
1930	2891	20,113	2980

As shown in Table 2, the training times for ResNet and Ensemble CNN are similar and approximately 5 times larger than for the proposed multi-threshold binarization. The same difference is observed for the inference time, where the proposed approach is able to classify the image for 0.08 s using 3.6 GHz Intel Core i5 CPU and Nvidia GTX 1060 GPU.

Finally, we compare the accuracy of the studied models with respect to the training time, which is required to achieve it. According to the results in Fig. 6, we can observe that with the short training time, multi-threshold binarization has up to 19% higher classification accuracy compared to ResNet and Ensemble CNN models. Both CNN-based models are able to show decent results after a significantly longer training time. Note that within the current experimental setup, we do not have the results for multi-threshold binarization for the longer training time, but even with a short training time, it seems to lose only 3–5% compared to the well-trained CNN counterparts. Thus, the proposed multi-threshold binarization can be considered a good alternative to the complex and deep CNN architectures for real-time remote sensing applications.

## Conclusion

In this paper, we have proposed a new solution to tackle the real-time image classification problem for remote sensing applications. In the proposed approach the typical deep CNN feature extractor is replaced by the multi-threshold binarization, which allows us to obtain highly discriminative features with much lower computational complexity. Experimental results on the TipJul1 and Zurich datasets show that the proposed multi-threshold binarization provides better classification accuracy after training on small datasets, while being outperformed by ResNet and Ensemble CNN on larger datasets. Nevertheless, in terms of complexity, the proposed approach provides 5 times lower training and inference time compared to ResNet and Ensemble CNN while maintaining the classification accuracy. In our future research, we will compare how the MobileNet, ShuffleNet and proposed approach perform on the highly complex multi-spectral images with very large number of channels, and also much higher number of classes and samples.

## Data Availability

The datasets generated and analysed during the current study are available in the Landsat Collections repository, available at https://www.usgs.gov/landsat-missions/landsat-collections, and the HYPERLABELME repository, available at https://hyperlabelme.uv.es/.
